# Comparative study of absorption in tilted silicon nanowire arrays for photovoltaics

**DOI:** 10.1186/1556-276X-9-620

**Published:** 2014-11-18

**Authors:** Md Imrul Kayes, Paul W Leu

**Affiliations:** 1 Department of Industrial Engineering, University of Pittsburgh, Pittsburgh, PA 15261, USA

**Keywords:** Silicon, Nanowire, Photovoltaics, Light trapping

## Abstract

Silicon nanowire arrays have been shown to demonstrate light trapping properties and promising potential for next-generation photovoltaics. In this paper, we show that the absorption enhancement in vertical nanowire arrays on a perfectly electric conductor can be further improved through tilting. Vertical nanowire arrays have a 66.2% improvement in ultimate efficiency over an ideal double-pass thin film of the equivalent amount of material. Tilted nanowire arrays, with the same amount of material, exhibit improved performance over vertical nanowire arrays across a broad range of tilt angles (from 38° to 72°). The optimum tilt of 53° has an improvement of 8.6% over that of vertical nanowire arrays and 80.4% over that of the ideal double-pass thin film. Tilted nanowire arrays exhibit improved absorption over the solar spectrum compared with vertical nanowires since the tilt allows for the excitation of additional modes besides the HE _1*m*_ modes that are excited at normal incidence. We also observed that tilted nanowire arrays have improved performance over vertical nanowire arrays for a large range of incidence angles (under about 60°).

## Background

Much solar cell research has focused on silicon (Si) nanowires, which have been demonstrated to be a promising active layer material for next-generation solar cells [1-10]. Nanowires may orthogonalize light absorption and carrier collection processes to facilitate high optical absorption and efficient collection of photogenerated carriers [11]. Furthermore, nanowires have demonstrated light trapping properties, where their absorption is enhanced over that of planar Si [1-3,9]. These structures may also be deposited on low-cost or flexible substrates using chemical vapor deposition or contact transfer methods [12]. Various structures that break the symmetry of nanowire arrays such as nanocones [13-15] or aperiodic vertical arrays [16] have been demonstrated to have increased absorption over vertical nanowire arrays.

Tilting vertical nanowire arrays, which may be fabricated by a wet chemical etching with dry metal deposition method [17], may be an additional and simple way to improve their performance. In this paper, we investigate the optical performance of tilted nanowire arrays on a metal contact and compare their performance to that of vertical nanowire arrays. We systematically study the performance with regard to tilt angle and report how the nanowire tilt may be used to improve solar absorption and thus ultimate efficiency. While these two geometries have been compared previously in experiments [17], these comparisons have not been performed for structures with the same Si volume. We also demonstrate that this enhancement occurs over a broad range of incidence angles.

## Methods

**Figure 1:**
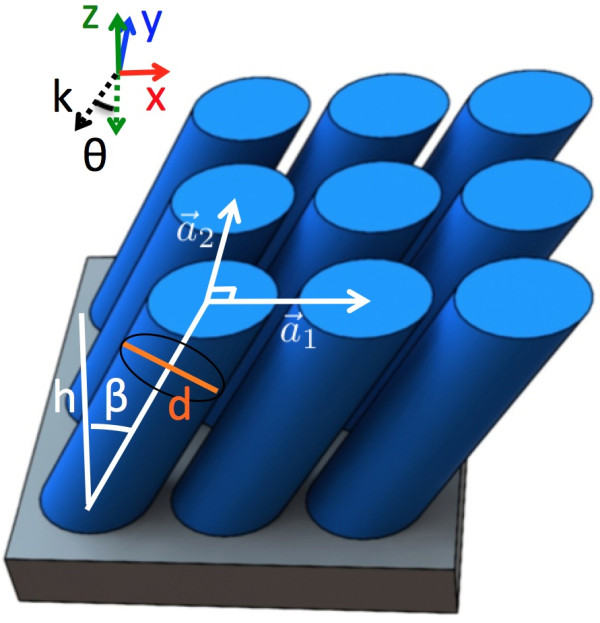
**Schematic of the tilted silicon nanowire array structure.** The nanowires sit on a perfectly electric conductor and are defined by the height *h*, circular cross-sectional diameter *d*, and tilt with respect to the zenith *β*. The nanowires form a two-dimensional lattice defined by the lattice vectors ${\vec{a}}_{1}$ and ${\vec{a}}_{2}$, where $|{\vec{a}}_{2}|=a$ and $|{\vec{a}}_{1}|=a/\text{cos}\beta$. The incidence angle of the incoming radiation is defined with respect to the negative *z*-direction.

Figure 1 shows a schematic of the tilted Si nanowire arrays studied, which sit on top of a perfectly electric conductor (PEC). The PEC may be, for example, the idealized perfectly reflecting back contact of a solar cell. The parameters of the structure are the height *h*, circular cross-sectional diameter *d*, and tilt with respect to the zenith *β*. The nanowires form a two-dimensional lattice defined by the lattice vectors ${\vec{a}}_{1}$ and ${\vec{a}}_{2}$, where ${\vec{a}}_{1}$ and ${\vec{a}}_{2}$ are in the *x*- and *y*-directions, respectively. The nanowires are tilted in the *x*-*z* plane. $|{\vec{a}}_{2}|=a$ and $|{\vec{a}}_{1}|=a/\;\text{cos}\beta$, where *a* is the vertical nanowire pitch. The total Si nanowire array volume is invariant with *β* since the fill factor $\left(\frac{\pi d^{2}}{4a^{2}}\right)$ is independent of *β*. This allows us to compare the performance of the nanowires as a function of tilt without changing the amount of Si. *d *≤ *a* in order to avoid the intersection of nanowires. We studied nanowires with *h *= 1,000 nm.

Maxwell’s equations were solved using the finite difference time domain (FDTD) method, which computes the energy-dependent transmission *T *(*E*), reflection *R *(*E*), and absorption spectra *A *(*E*) efficiently. The ultimate efficiency is calculated using the following equation:

(1) $$\eta =\frac{\overset{\infty }{\underset{E_{g}}{\int }}I(E)A(E)\frac{E_{g}}{E}\text{dE}}{\overset{\infty }{\underset{0}{\int }}I(E)\text{dE}},$$

where *E* is the photon energy, *E*_g_ is the bandgap of crystalline Si, *I *(*E*) is the solar irradiance under the global 37° tilt air mass 1.5 spectrum [18], and *A *(*E*) is the absorption [19]. The ultimate efficiency is the maximum efficiency of a solar cell when the temperature approaches 0 K, where there is no recombination and each absorbed photon produces an electron-hole pair. The bandgap *E*_g _= 1.12 eV for crystalline Si. The absorption and reflection spectra were obtained over the energy range of the solar spectrum from *E *= 1.12 to 4.13 eV (wavelengths from 1,100 to 300 nm). Assuming each photon produces an electron-hole pair and there is no recombination, such that all photogenerated carriers are collected, the short-circuit current density is

(2) $$J_{\text{sc}}=q\overset{\infty }{\underset{E_{g}}{\int }}b_{s}(E)A(E)\text{dE},$$

where *q* is the elementary charge and *b*_*s*_(*E*) is the photon flux density. The irradiance and photon flux density are related by *I *(*E*) = *Eb*_*s*_(*E*). The total solar absorption is calculated from

(3) $$A_{\text{sol}}=\frac{\overset{\infty }{\underset{E_{g}}{\int }}b_{s}(E)A(E)\text{dE}}{\overset{\infty }{\underset{0}{\int }}b_{s}(E)\text{dE}}.$$

The optical constants for Si were taken from experimental measurement results in Palik’s *Handbook of Optical Constants of Solids*[20]. A non-uniform mesh with a minimum size of 15 nm was used for the simulation. Perfectly matched layer boundary conditions were used for the upper boundary of the simulation cell [21], PEC boundary conditions were used for the lower boundary of the simulation cell, and appropriate boundary conditions were used for the side boundaries to model the periodic nature of the arrays.

## Results and discussion

**Figure 2:**
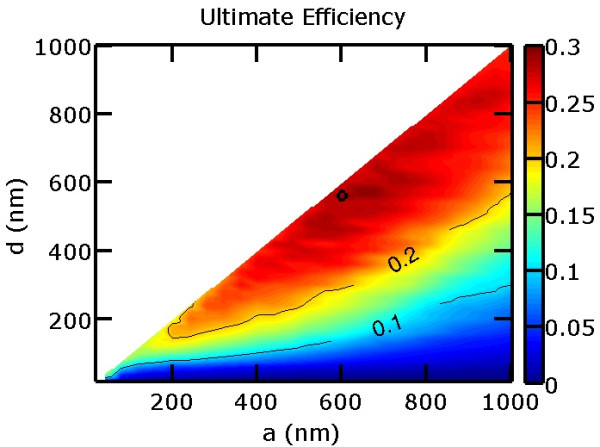
**Ultimate efficiency of square arrays of vertical nanowires.** The vertical nanowires (*β *= 0) have height *h *= 1,000 nm, and the ultimate efficiency is shown as a function of array pitch *a* and nanowire diameter *d*. The highest ultimate efficiency is 29.7% at *a *= 600 nm and *d *= 560 nm, which is marked with a circle in the contour plot.

We first focused on evaluating vertical nanowires where *β *= 0. The vertical nanowires form a square lattice with $|{\vec{a}}_{1}|=|{\vec{a}}_{2}|=a$. *h*= 1,000 nm as mentioned before. We varied the pitch *a* from 40 to 1,000 nm and the diameter *d* from 20 nm up to the pitch. Figure 2 shows the ultimate efficiency as a function of pitch and diameter. The best vertical nanowire array was determined to be with *a *= 600 nm and *d *= 560 nm, which is marked with a circle in the contour plot. The pitch of 600 nm is the same as that found in previous research [2,22]. High efficiencies are generally achievable from arrays with high fill factors. The best vertical nanowire has an ultimate efficiency of 29.7%.

The vertical nanowire array with *a *= 600 nm and *d *= 560 nm has the same amount of Si as a flat thin film structure with a thickness of *L *= 684 nm. For comparison purposes, we also calculated the absorption spectrum of a double-pass thin film with the same amount of Si. The double-pass thin film assumes perfect antireflection at the front surface of the thin film and perfect reflection at the back surface, such that light passes through the material twice with no light trapping. The absorption for double-pass thin films under normal incidence light is

(4) $$A(E)=1-\text{exp}\left[-2\alpha (E)L\right],$$

where *α *(*E*) is the energy-dependent absorption coefficient of Si. The ultimate efficiency of an ideal double-pass thin film with *L *= 684 nm is 17.9%.

**Figure 3:**
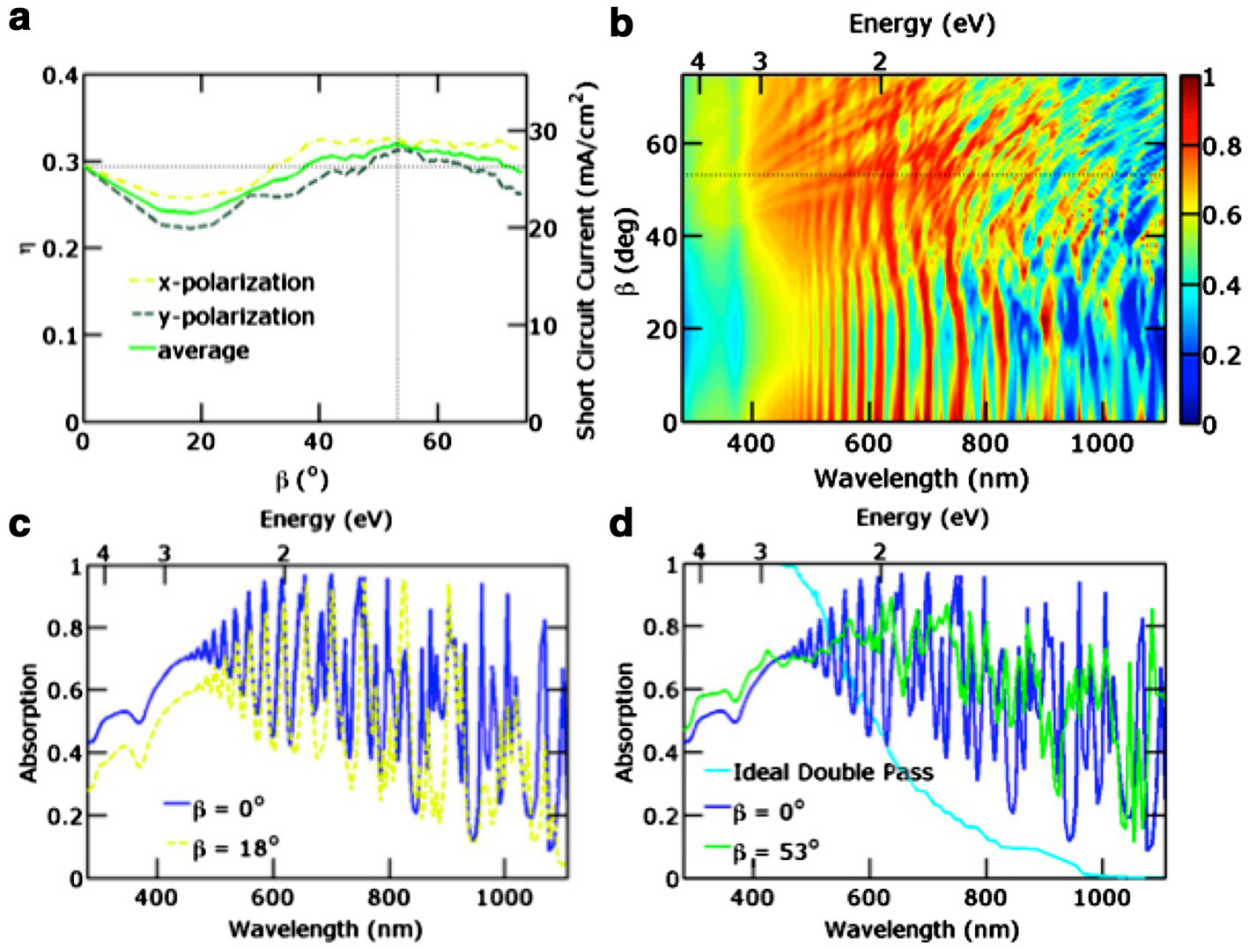
**Tilted nanowire array simulation results.** The nanowires have height *h *= 1,000 nm, diameter *d *= 560 nm, and vertical pitch *a *= 600 nm. **(a) ** Ultimate efficiency *η* function of nanowire array tilt angle *β*. The short-circuit current density is shown on the right *y*-axis. **(b) ** Absorption as a function of wavelength and nanowire tilt *β*. The absorption shown is the average of the two polarizations. **(c)** The absorption spectra of the vertical nanowire array (*β *= 0°) and worst tilted nanowire array (*β *= 18°). **(d)** The absorption spectra of the ideal double-pass thin film, vertical nanowire array, and best tilted nanowire array (*β *= 53°).

Next, we studied tilted nanowire arrays. The pitch and diameter were fixed at *a *= 600 and *d *= 560 nm respectively, while the nanowires were systematically tilted by varying *β*. The height is fixed at *h *= 1,000 nm. As mentioned earlier, the amount of Si does not change as the wires are tilted. The simulations were performed with the normal incident light polarized in the *x*-direction and then in the *y*-direction as the nanowires are tilted. Figure 3 plots the results of our tilted nanowire array studies. Figure 3a plots the ultimate efficiency *η* as a function of the nanowire array tilt angle *β*. *η *= 29.7% for the vertical nanowire array (*β *= 0°). A horizontal dotted line is shown at this ultimate efficiency for reference. The ultimate efficiency is shown for incident light polarized in the *x*-direction, *y*-direction, and the average of these two results.

By tilting the nanowires, higher average ultimate efficiencies may be achieved at nanowire array tilts between 38° and 72°. The ultimate efficiency is 32.2% and a maximum at *β*= 53°, which is indicated with a vertical dotted line. ${\vec{a}}_{1}=997$ nm at this tilt angle. Figure 3b plots the absorption as a function of the wavelength and nanowire tilt angle *β*. The absorption shown is the average of the two polarizations. The optimum tilt of *β *= 53° is again marked with a dashed line in the contour plot. Normal incident light can only couple to HE _1*m*_ in vertical nanowires due to symmetry requirements [23]. Distinct resonance peaks can be seen in the absorption spectrum of the vertical nanowire array. For tilts less than 38°, the ultimate efficiency decreases when compared to the vertical nanowire arrays. The minimum ultimate efficiency is 24.0% at *β *= 18°. Figure 3c plots the absorption spectra of the vertical nanowire array (*β *= 0°) and this tilted nanowire array. The absorption spectra of the *β *= 18° tilted nanowire array closely resembles that of the vertical nanowire array, but the magnitude of the various absorption resonances is lower. For small tilt angles, the ultimate efficiency decreases because the excitation efficiencies of the HE _1*m*_ modes decrease.

However, as the nanowire is tilted, additional modes besides the HE _1*m*_ modes may be excited. The overall absorption for tilted nanowires is thus increased compared to tilted nanowires for higher tilt angles. The tilted nanowires exhibit improved performance compared to vertical nanowires over the range of *β *= 38° to 72°. Figure 3d plots the absorption spectra of the ideal double-pass thin film, vertical nanowire array, and best tilted nanowire array (*β *= 53°). The absorption spectra of the tilted nanowire is broader with less distinct resonance peaks. Table 1 lists the fraction of photons absorbed in different regions of the solar spectrum for these three. The infrared region (above the Si bandgap) is from 1.12 to 1.67 eV (1,100 to 740 nm), the visible region is from 1.67 to 3.1 eV (740 to 400 nm), and the ultraviolet region is from 3.1 to 4.4 eV (400 to 280 nm). The total absorption shown only includes the range above the c-Si bandgap energy from 1.12 to 4.4 eV (1,100 to 280 nm). Vertical nanowire arrays have improved absorption in the lower energy regime (infrared and visible regions) compared with the ideal double-pass Si thin film due to light trapping. Tilted nanowire arrays improve the light trapping performance even more by increasing absorption across the infrared, visible, and ultraviolet regions compared to the vertical nanowire arrays.

**Table 1:** Absorption (%) in different spectral regions

**Spectrum**	**IR**	**Vis**	**UV**	**Total**
Ideal double pass	6.0	59.4	100.0	36.4
Vertical nanowire array	41.8	70.5	57.2	60.5
Tilted nanowire array	46.7	74.7	60.0	65.7

The infrared and total solar regions are calculated for those portions that are above the Si bandgap (*E ****> *** 1.12 eV).

The performance of these three different structures for solar cells are compared in Table 2. The total solar absorption, ultimate efficiency, and short-circuit current density are all shown in this table. The total solar absorption shown in this table is the absorption over the entire solar spectrum and not just over energies above the Si bandgap as shown in Table 1. The ultimate efficiency of the vertical nanowire arrays is 29.7%, a 66.2% improvement over the 17.9% exhibited by the ideal double-pass thin film with the equivalent amount of material. The ultimate efficiency of the tilted nanowires arrays is 32.2%, or an improvement of 8.6% compared to vertical nanowire arrays or 80.4% over the ideal double-pass thin film. These simulations demonstrate the potential of tilted nanowire arrays to improve the performance of vertical nanowire arrays under normal incidence.

**Table 2:** **The total solar absorption (*A***_**sol**_**), short-circuit current density (*J***_**sc**_**), and the ultimate efficiency (*η*)**

	***A***_**sol**_** (*%*)**	***J***_**sc**_** (mA/cm **^**2**^**)**	***η* (%)**
Ideal double pass	23.1	15.9	17.9
Vertical nanowire array	38.4	26.5	29.7
Tilted nanowire array	41.7	28.8	32.2

**Figure 4:**
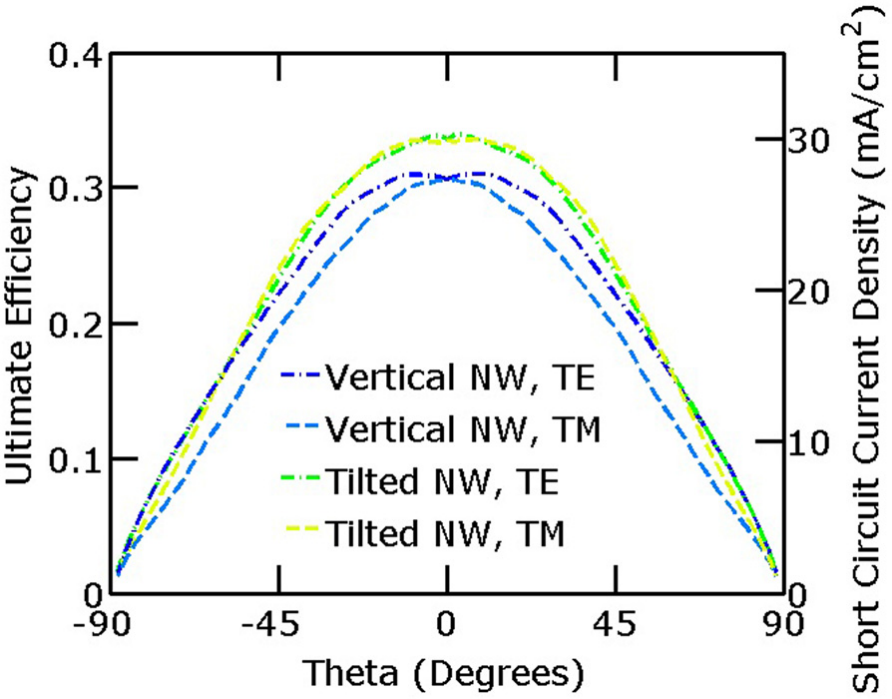
**Ultimate efficiency of vertical nanowire and tilted nanowire arrays as a function of incidence angle *θ*.** The TE polarization results for the tilted nanowire arrays are the average of the light polarized along the *x*-axis and the *y*-axis. Likewise, the TM polarization results for the tilted nanowire arrays are the average of the magnetic field along the *x*-axis and the *y*-axis. The tilted nanowire arrays consistently perform better than the vertical nanowire arrays for incidence angles under about 60°.

We further studied the performance of the nanowire arrays under oblique incidence. The source was varied from *θ *= - 90° to 90° for the best vertical nanowire array (*a *= 600 nm, *d *= 560 nm, and *β *= 0°) and the best tilted nanowire array (*a *= 600 nm, *d *= 560 nm, and *β *= 53°). The incidence angle *θ* is with respect to the negative *z*-direction as shown in the Figure 1 schematic. Figure 4 shows the results for transverse electric (TE) waves, which are linearly polarized transverse to the plane of incidence, and transverse magnetic (TM) waves, which have linear polarization so that the magnetic field is purely transverse. The results at an incidence angle of *θ *= 0° are slightly different from that above due to the use of different boundary conditions.

The performance of the vertical nanowire arrays is symmetric with respect to positive and negative incidence angles. While freestanding vertical nanowire arrays have better performance under TM-polarized incident light than TE [2,8], our results indicate that vertical nanowire arrays on a perfect back reflector have the opposite trend where the ultimate efficiency is higher for TE polarization than TM polarization. The increased absorption under TM-polarized incident light in freestanding vertical nanowires is due to reduced transmission [24], whereas our system has no transmission due to the perfect back reflector.

In our simulations of tilted nanowires, the performance is symmetric with respect to positive and negative incidence angles for TE waves with electric field along the *x*-axis and TM waves with magnetic field along the *y*-axis. However, this symmetry is broken for TE incidence with electric field along the *y*-axis and TM incidence with magnetic field along the *x*-axis. The results shown for the tilted nanowire arrays are the average of the two orthogonal polarizations. The performance of the tilted nanowires is slightly better under positive incidence angles versus negative incidence angles, where the Poynting vector is closer to along the axis of the nanowire. In addition, the performance of the tilted nanowires is consistently higher than that of the vertical nanowire arrays for incidence angles under about 60°. For high angles of incidence, the performance of the vertical and tilted nanowires converge for both TE incidence and TM incidence.

## Conclusions

We have performed a comparative study of the optical performances of tilted Si nanowire arrays on a perfectly electric conductor for photovoltaic applications using the finite difference time domain method. Our results show that the absorption enhancement in vertical nanowire arrays over Si thin films can be further improved through tilted nanowires. Optimized vertical nanowire arrays with a height of 1,000 nm have a 66.2% ultimate efficiency improvement over an ideal double-pass thin film of the equivalent amount of material. Tilted nanowire arrays, with the same amount of material, exhibit improved performance compared to vertical nanowires arrays over a broad range of tilt angles (from 38° to 72°). The optimum tilt of 53° has an improvement of 8.6% over that of the vertical nanowire arrays and 80.4% of the ideal double-pass thin film. Tilted nanowire arrays exhibit improved absorption over the infrared, visible, and ultraviolet regimes compared with vertical nanowires since the tilt allows for the excitation of additional modes besides the HE _1*m*_ modes that are excited at normal incidence. We also observed that tilted nanowire arrays have improved performance over vertical nanowire arrays over a large range of incidence angles (under about 60°).

## Competing interests

The authors declare that they have no competing interests.

## Authors’ contributions

MIK participated in the design of the study, carried out simulations, and drafted the manuscript. PWL supervised the project, participated in the design of the study and analysis of its results, and revised the manuscript. Both authors read and approved the final manuscript.
